# A Novel Live Attenuated Vaccine Candidate Protects Against Heterologous *Senecavirus A* Challenge

**DOI:** 10.3389/fimmu.2019.02660

**Published:** 2019-11-26

**Authors:** Bishwas Sharma, Maureen H. V. Fernandes, Marcelo de Lima, Lok R. Joshi, Steve Lawson, Diego G. Diel

**Affiliations:** ^1^Animal Disease Research and Diagnostic Laboratory, Department of Veterinary and Biomedical Sciences, South Dakota State University, Brookings, SD, United States; ^2^Center for Biologics Research and Commercialization, South Dakota State University, Brookings, SD, United States; ^3^Department of Population Medicine and Diagnostic Sciences, Animal Health Diagnostic Center, College of Veterinary Medicine, Cornell University, Ithaca, NY, United States; ^4^Laboratório de Virologia e Imunologia Animal, Faculdade de Veterinária, Universidade Federal de Pelotas, Pelotas, Brazil

**Keywords:** *Senecavirus A*, Seneca Valley virus, recombinant virus, live attenuated vaccine, inactivated vaccine

## Abstract

*Senecavirus A* (SVA) is an emerging picornavirus causing vesicular disease (VD) clinically indistinguishable from foot-and-mouth disease (FMD) in pigs. Currently there are no vaccines currently available for SVA. Here we developed a recombinant SVA strain (rSVAm SacII) using reverse genetics and assessed its immunogenicity and protective efficacy in pigs. *In vivo* characterization of the rSVAm SacII strain demonstrated that the virus is attenuated, as evidenced by absence of lesions, decreased viremia and virus shedding in inoculated animals. Notably, while attenuated, rSVA mSacII virus retained its immunogenicity as high neutralizing antibody (NA) responses were detected in inoculated animals. To assess the immunogenicity and protective efficacy of rSVA mSacII, 4-week-old piglets were sham-immunized or immunized with inactivated or live rSVA mSacII virus-based formulations. A single immunization with live rSVA mSacII virus via the intramuscular (IM) and intranasal (IN) routes resulted in robust NA responses with antibodies being detected between days 3–7 pi. Neutralizing antibody responses in animals immunized with the inactivated virus via the IM route were delayed and only detected after a booster on day 21 pi. Immunization with live virus resulted in recall T cell proliferation (CD4^+^, CD8^+^, and CD4^+^/CD8^+^ T cells), demonstrating efficient stimulation of cellular immunity. Notably, a single dose of the live attenuated vaccine candidate resulted in protection against heterologous SVA challenge, as demonstrated by absence of overt disease and reduced viremia, virus shedding and viral load in tissues. The live attenuated vaccine candidate developed here represents a promising alternative to prevent and control SVA in swine.

## Introduction

*Senecavirus A* (SVA) is a vesicular disease (VD)-causing pathogen of pigs and the only species of the genus *Senecavirus* in the family *Picornaviridae* (1). SVA is a non-enveloped, icosahedral virus with a single-stranded positive sense RNA genome with ~7.2 kb. The SVA genome encodes a unique open reading frame (ORF), which is proteolytically processed in four structural proteins (VP1-VP4) and eight non-structural proteins (L, 2A−2B−2C−3A−3B−3C−3D) (2). The virus genome is organized in a central coding region (ORF1) flanked by 5′- and 3′-untranslated regions (UTRs) and a poly(A) tail following the 3′-UTR (2).

*Senecavirus A* was first identified as a contaminant of human fetal retinal cells (PER.C6) in the US in 2002 (3). Retrospective sequencing of archived picorna-like viruses at the United States Department of Agriculture National Veterinary Service Laboratories (NVSL), revealed the circulation of SVA in the US swine population since at least 1988 (3). Since its first description in 2002, SVA has been explored as an oncolytic agent for cancer treatment in humans (4–6). Recently the virus gained importance in the veterinary field due to the increased incidence of SVA-induced VD in pigs. Since 2014, SVA has been associated with VD outbreaks in swine in Canada (7), the US (2, 8–10), Brazil (9, 11, 12), Colombia (13), China (14), Thailand (15), and Vietnam (16). Pigs are thought to be the main reservoir for SVA; however, the virus has been also isolated from mice, and its nucleic acid has been detected in houseflies collected in SVA affected and non-affected farms (9). Additionally, neutralizing antibodies against SVA have been detected in pigs, cattle and mice (3). The importance of these species for the epidemiology of SVA, however, remains unknown. The protein Anthrax Toxin Receptor 1 (ANTXR1) has been identified as a potential receptor for SVA and shown to interact with the virus capsid during infection of human H446 cancer cells (17), however, the contribution of this molecule to SVA infection in swine await experimental confirmation.

The clinical relevance of SVA, lies on its similarity with other high-consequence VDs of swine, including FMD, swine vesicular disease (SVD), vesicular stomatitis (VS) and vesicular exanthema of swine (VES) (12). Infection with SVA likely occurs via the oral and/or respiratory routes and after an incubation period of 3–5 days, clinical signs including lethargy and lameness are observed. The clinical signs are followed by development of vesicles on the snout and/or feet (dewclaw, interdigital space coronary band and sole) of affected animals (18). The lesions are characterized by cutaneous hyperemia which progresses into fluid-filled vesicles. As the disease progresses, the vesicles rupture and evolve into skin erosions that eventually scab and resolve within 12–16 days post-infection (pi) (18–20). A short-term viremia (1–10 days post-infection, pi) occurs in infected animals and the levels of viremia decline as serum neutralizing antibody (NA) levels rise (19).

The immune responses to SVA are characterized by the development of early and robust NA titers (18, 19, 21), which are strongly correlated with VP2- and VP3-specific IgM responses within the first week of infection (19). Notably, NA levels parallel with decreased viremia and resolution of the disease (19). Analysis of the major porcine T cell subsets revealed that during the acute/clinical phase of SVA infection (14 days pi) T cell responses are characterized by an increased frequency of αβ T cells, especially CD4^+^ T cells that are initially detected by day 7 pi and increase in frequency until day 14 pi. Additionally, the frequency of CD8^+^ and double-positive CD4^+^CD8^+^ T cells (effector/memory T cells) expressing IFN-γ or proliferating in response to recall SVA stimulation increases after day 10 pi (19). These observations indicate that SVA elicits B and T cell activation early upon infection, with IgM antibody levels being associated with early neutralizing activity against the virus and peak B and T-cell responses paralleling with clinical resolution of the disease, suggesting that both arms of the immune system may contribute to the control of SVA infection. Importantly, there is only one known serotype of SVA (2, 22) and genetically diverse viral strains present cross neutralizing- and cross T-cell responses (22).

Currently there are no vaccines available for SVA. A recent study assessed the immunogenicity of an inactivated SVA vaccine candidate, demonstrating protection against homologous virus challenge (23). The most effective picornavirus vaccines consist of inactivated or live attenuated vaccines. Most vaccines used for FMDV, for example, are inactivated; however, they fail to induce long-term protection requiring annual re-vaccinations (24). Poliovirus (PV) vaccines long used in humans, leading to the eradication of wild type poliovirus, were either inactivated poliovirus vaccine (IPV) or live oral poliovirus vaccine (OPV). The live OPV induces long-lasting mucosal immunity against PV. In this study we generated a recombinant attenuated SVA strain based on a contemporary SVA isolate (18) and assessed the immunogenicity and protective efficacy of inactivated or live virus formulations against heterologous SVA challenge.

## Materials and Methods

### Viruses and Cells

H1299 and BHK-21 cells were obtained from the American Type Culture Collection (ATCC-CRL 5803 and CCL-10, respectively). PK-15 cells were obtained from the Virology section at the Animal Disease Research and Diagnostic Laboratory (ADRDL). H1299 and PK-15 BHK-21 cells were maintained at 37°C with 5% CO_2_ in RPMI-1640 or MEM (Corning, NY), respectively, supplemented with 10% fetal bovine serum (VWR, Chicago, IL) and 2 mM L-Glutamine (Corning, NY). Penicillin (100 U/mL) and streptomycin (100 μg/mL) were also added to culture media.

SVA strain SD15-26 was isolated from swine presenting vesicular disease and has been previously characterized (18, 19). The challenge virus, SVA strain MN15-84-22, was isolated from swine presenting vesicular disease (9). For both wild type SVA strains, low-passage (passage 4) viral stocks were amplified and titrated in H1299 cells and used in experiments described below. Recombinant rSVA mSacII virus was rescued in BHK-21 and H1299 cells and amplified in PK15 cells as described below.

### Generation, Rescue, and Amplification of rSVA mSacII Virus

An infectious virulent cDNA clone of SVA strain SD15-26 (pBrick-FLSVA-SD15-26) containing the full length SVA genome under control of a T7 RNA polymerase promoter was recently developed in our laboratory (Fernandes et al., unpublished data). The pBrickA-FLSVA-SD15-26 was constructed using a strategy combining: i. synthesis of cDNA fragments corresponding to the 5′- and 3′ ends of the SVA genome and ii. PCR amplification of the central region of the SVA genome, followed by restriction digestion and cloning. The clone was used as a backbone to construct the rSVA mSacII virus here. In the rSVA mSacII clone, four nucleotide changes were introduced in the virus genome. Three of those changes are in the 5′UTR (c→t, positions 29, 31 and 32) and the fourth change consists of a silent nucleotide change (c→a) at position 942 (VP4 coding region) of the rSVA genome (added to delete a SacII restriction endonuclease site). A synthetic DNA fragment (GenScript) containing these specific nucleotide changes was cloned into the backbone of the rSVA plasmid (virus described above; pBrick-FLSVA-SD15-26) using unique restriction endonucleases (NheI and SfiI) and standard cloning techniques. The resultant recombinant SacII mutant clone (pBRICK-rSVA mSacII) was amplified in Stable 2 cells (Life Technologies) and the purified plasmid DNA was linearized with NotI-HF (NEB) restriction enzyme, which digests the cDNA clone after the poly A tail. The linear pBRICK-rSVA mSacII DNA was used as template in *in vitro* transcription reactions using the MEGAscript™ T7 Transcription Kit (ThermoFisher Scientific) following the manufacturer's instructions. Full-length viral genomic RNA was purified using standard phenol:chloroform purification and ethanol precipitation, and ~1 μg of viral RNA was transfected in BHK-21 cells using Lipofectamine™ RNAiMAX transfection reagent (ThermoFisher Scientific) according to the manufacturer's instructions. At 48 h post-transfection cells were subjected to three freeze-and-thaw cycles and the virus passaged two more times in H1299 cells. When cytopathic effect was observed, cells were fixed and rescue of rSVA mSacII virus was confirmed by immunofluorescence (IFA) using a rabbit polyclonal antibody against SVA.

The identity of the rSVA mSacII virus was confirmed by RT-PCR amplification of a fragment in the 5′ end of the SVA genome (primers sequences available upon request) followed by SacII restriction digestion of the PCR amplicon. Restriction digestion reactions were analyzed by agarose gel electrophoresis and the identity of rSVA mSacII was determined by lack of SacII digestion in the rSVA mSacII virus PCR amplicon. A PCR amplicon of wt rSVA SD15-26 was used as control. Additionally, complete genome sequencing of rSVA mSacII virus was used to confirm the identity and integrity of the virus genome using the Illumina MiSeq sequencing platform (18). Stocks of rSVA mSacII (p. 4) were produced in PK15 cells. Semi-confluent monolayers of PK15 cells were inoculated with a low MOI (~0.1) of rSVA mSacII and incubated at 37°C for 72 h until complete SVA CPE was observed in inoculated monolayers. Cells were subjected to three freeze-and-thaw cycles and virus stocks were cleared by centrifugation, aliquoted (1 ml), and stored at −80°C until used in the experiments described below.

### Growth Curves

Replication kinetics of wt SVA SD15-26 and rSVA mSacII were assessed *in vitro*. H1299 cells were cultured in six-well plates, infected with both viruses at a multiplicity of infection (MOI) of 0.1 (multi-step growth curve) or 10 (single-step growth curve), and harvested at various time points post-infection (2, 4, 8, 12, and 24 h post-infection). Virus titers were determined using end-point dilution and the Spearman and Karber's calculation method and expressed as TCID_50_/ml.

### Western Blots

Western blot was performed to assess protein expression by the rSVA mSacII virus in comparison with wt SVA virus. For this, H1299 cells were infected with both viruses at a MOI of 10 and harvested at different time points post-infection (0, 2, 4, 8, 12, and 24 h). Cells were lysed with M-PER mammalian protein extraction reagent (ThermoFisher Scientific) containing protease inhibitors. Approximately, 100 μg total protein extracts were mixed with Laemmli Buffer (Bio-Rad, Hercules, CA) containing 5% β-mercaptoethanol and denatured at 95°C for 10 min then loaded in 10% SDS-PAGE gel. Electrophoresis was performed at 90 volts for 90 min and proteins were transferred to nitrocellulose membranes and blocked with 5% skim-milk in 1 × phosphate-buffered saline (PBS) overnight at 4°C. Nitrocellulose membranes were washed three times in 1 × PBS containing 0.05% Tween 20 (PBST) and incubated with anti-VP1 and anti-VP2 mouse monoclonal antibodies (kindly provided by Dr. Steve Lawson, SDSU) (1:1,000) in 0.05% PBST for 2 h at RT. Membranes were washed three times with 0.05% PBST. Secondary IRDye® 800CW Goat anti-Mouse IgG (H+L) (LI-COR Biosciences, Lincoln, NE) antibody was added to the membranes (1:15,000 on 1% skim-milk on 0.05% PBST) and incubated for 1 h at RT. Membranes were washed three times with PBST 0.05% and blots developed using a LI-COR® Odyssey® Fc Imaging system (LI-COR Biosciences, Lincoln, NE).

The antigen load in both inactivated and live rSVA mSacII vaccine formulations was also assessed by western blots. For this, 20 μL of each vaccine formulation containing the rSVA mSacII virus suspension at 10^6^ TCID_50_/mL were mixed with Laemmli Buffer (Bio-Rad, Hercules, CA) and subjected to SDS-PAGE and immunoblotting as described above.

### Animal Pathogenesis Study

The pathogenicity of the rSVA mSacII virus was investigated in pigs. For this, twelve 15-week old SVA-negative finishing pigs weighing ~60 kg were randomly allocated in two experimental groups as follows: Group 1, wt SVA SD15-26- inoculated group (*n* = 6), and rSVA mSacII-inoculated group (*n* = 6). Animals from both groups were inoculated with virus suspensions containing 10^8.5^ TCID_50_ via the oronasal route (5 mL orally and 5 mL intranasally [half into each nostril]). Animals were challenged on arrival at SDSU Animal Resource Wing (ARW). Animals received food and water *ad libitum* for the duration of the 14-day experiment.

Animals were monitored daily after inoculation for characteristic SVA clinical signs and lesions. Clinical signs and lesions were recorded, and individual daily lesion scores were attributed to each animal (22) and total daily scores were calculated. Swabs (oral, nasal and rectal) and blood samples (serum and whole heparinized blood) were collected on days 0, 1, 3, 7, 10, and 14 pi. At necropsy on day 14 pi, tissues including heart, lungs, kidney, liver, small intestine, large intestine, thymus, spleen, mediastinal lymph node, mesenteric lymph node, and tonsil were collected and stored at−80°C. Animal experiments were revised and approved by the SDSU Institutional Animal Care and Use Committee (approval number 16-002A).

### Immunization-Challenge Experiment

The immunogenicity and protective efficacy of rSVA mSacII virus were evaluated in 3-week-old SVA-negative piglets. Animals were randomly allocated in four experimental groups: control (G1, received RPMI 1640, IM; *n* = 6), inactivated (G2, received BEI-inactivated rSVA mSacII vaccine; *n* = 6), live IM (G3, receiving rSVA mSacII by IM route; *n* = 6) and live IN (G4, received rSVA mSacII by IN route; *n* = 6). After a week of acclimation, animals were immunized with the corresponding candidate vaccines (2 mL) via the immunization routes described above and presented in Table 1. Inactivation of rSVA mSacII virus by BEI was performed as previously described (25), and a water-in-oil-in-water (W/O/W) emulsion was produced by shear-mixing equal volume of the MONTANIDE^TM^ ISA 201 VG oil adjuvant (1 mL, Seppic SA, Paris) with BEI inactivated virus (1 mL; 10^6^ TCID_50_) at 31°C using syringes joined by a lure lock connector as recommended by the adjuvant manufacturer. The live rSVA mSacII vaccine consisted of 2 ml virus suspension (10^6^ TCID_50_) in RPMI 1640 medium. Animals in control and inactivated groups were immunized on day 0 and boosted on day 21 post-primary immunization, whereas animals in the live IM and live IN groups were immunized with a single vaccine dose on day 0.

**Table 1:** Animal immunization/challenge experimental design.

**Group (*n*)**	**Treatment**	**Dose**	**Route**	**Immunization day**	**Virus challenge^**b**^**
1 (*n* = 6)	Control	2 mL RPMI	IM	0 and 21	SVA MN15-84-22 10^8.5^ TCID_50_ at 42 dpi
2 (*n* = 6)	Inactivated (BEI) SVA^a^	10^6^ TCID_50_ in 2 mL	IM	0 and 21	
3 (*n* = 6)	Live attenuated rSVA mSacII	10^6^ TCID_50_ in 2 mL	IM	0	
4 (*n* = 6)	Live attenuated rSVA mSacII	10^6^ TCID_50_ in 2 mL	IN	0	

*aAdjuvant: Seppic Montanide™ ISA 201 (1:1)*.

*bAnimals were challenged oronasally with 10 mL of virus inoculum (18)*.

Animals were monitored daily for signs and lesions throughout the experiment. Oral, nasal, and rectal swabs were collected on days 0, 3, 7, 14, and 21 pi. Blood was collected on days 0, 3, 5, 7, 14, 21, 28, and 35 pi. Serum separation and PBMC isolation were performed as previously described (19).

A heterologous SVA isolate (SVA MN15-84-22; 97% nt identity with SVA SD15-26) (9) was used as challenge virus and animals in all groups were challenged on day 42 pi (or day 0 post-challenge; pc). Blood and swabs (oral, nasal, and rectal) were collected on days 0, 3, 7, 10, and 14 pc and processed and stored as above. All animals were euthanized on day 14 pc at the Animal Disease Research and Diagnostic Laboratory (ADRDL), SDSU. Tissues including tonsil and mediastinal and mesenteric lymph nodes were collected and stored at −80°C. Animal immunization-challenge experiments were reviewed and approved by the SDSU Institutional Animal Care and Use Committee (approval number 18-032A).

### RNA Extraction and Real-Time Reverse Transcriptase PCR (RT-qPCR)

Nucleic acid was extracted from serum, swabs, and tissue samples using the Cador® Pathogen 96 kit (Indical Bioscience) and the QIAcube® HT (Qiagen) automated extractor following the manufacturer's instructions. Swab samples were vortexed and cleared by centrifugation (10,000 × g for 5 min) and 200 μL of cleared supernatant was used for nucleic acid extraction. Two hundred μL of serum were used for nucleic acid extraction. For tissues, ~0.5 g of each tissue was minced using sterile scalpel, re-suspended in RPMI 1640 medium (10% w/v) and homogenized using a stomacher (2 cycles of 60 s). Homogenized samples were then centrifuged at 14,000 × g for 2 min at room temperature and 200 μL of cleared supernatant was used for nucleic acid extraction using automated QIAcube HT (Qiagen). The presence of SVA RNA in samples was assessed using the SensiFAST^TM^ Probe LO-ROX One-Step kit (Bioline-Meridian Bioscience, MA, USA) and custom designed primers and probe (PrimeTime qPCR probe assays, Integrated DNA Technologies Inc., USA) targeting the SVA 3D gene. Primers and probe were designed using the PrimerQuest Tool (Integrated DNA Technologies Inc., USA). The probe and primers sequence are 5'-/56-FAM/CAGGAACAC/ZEN/TACTCGAGAAGCTGCAA/3IABkFQ/-3′, 5′- GAAGCCATGCTCTCCTACTTC-3′ and 5′- GGGTGCATCAATCTATCATATTCTTC-3′ respectively. Amplification and detection were performed with an Applied Biosystems 7500 real time PCR system under following conditions: 10 min at 45°C for reverse transcription, 2 min at 95°C for polymerase activation and 40 cycles of 5 s at 95°C for denaturation and 30 s at 60°C for annealing and extension. A standard curve was established by using a SVA SD15-26 virus suspension containing 10^7.88^ TCID50/mL and preparing 10-fold serial dilutions from 10^−1^ to 10^−10^. Relative viral genome copy numbers were calculated based on the standard curve determined using the four- parameter logistic regression model function within MasterPlex Readerfit 2010 software (Hitachi Software Engineering America, Ltd., San Francisco, CA). The amount of viral RNA detected in samples were expressed as log_10_ (genome copy number)/mL.

### Neutralization Assays

Neutralizing antibody (NA) responses elicited by the vaccine candidates and post-challenge (pc) infection were assessed using a virus neutralization assay as previously described (18, 19). Neutralization assays were performed using the parental SVA SD15-26 and the challenge MN15-84-22 virus. NA titers were expressed as log_2_ (reciprocal of highest serum dilution capable of completely inhibiting SVA infection). All assays were performed in triplicate and included positive and negative controls in all test plates.

### PBMC Recall Stimulation and Flow Cytometry

PBMC recall stimulation was performed as previously described (19). Briefly, PBMCs were thawed and stained with 2.5 μM carboxyfluorescein succinimidyl ester (CFSE; in PBS) according to the manufacturer's instruction (BD Biosciences). CFSE-stained cells were seeded at a density of 5 × 10^5^ cells/well in 96-well plates, rested for 4 h and stimulated as follows: UV-inactivated SVA SD15-26 [multiplicity of infection [MOI] = 2] and recombinant purified VP2 protein (1 μg/mL). Concanavalin A (ConA; 5 μg/mL) plus phytohemagglutinin (PHA; 5 μg/mL) (both from Sigma-Aldrich, St. Louis, MO), or cRPMI alone were used as positive and negative controls in all assays, respectively. After stimulation, the cells were incubated for 5 days at 37°C with 5% CO_2_.

Antigen-specific T-cell responses were assessed by flow cytometric analysis. T-cell phenotypes were determined using the various swine-specific antibodies as previously described (19). Single-stain and fluorescence-minus-one (FMO) controls were included in all assays. All flow cytometry data were acquired with an Attune NxT flow cytometer (Thermo Fisher Scientific) and analyzed using FlowJo v.10 software (TreeStar, San Carlos, CA). The percentage of responding cells was calculated as the percentage of total T cells (live CD3^+^ cells).

### Statistical Analysis

Statistical analysis was performed by analysis of variance (ANOVA) followed by Tukey's multiple comparison test. Normality was checked before performing any tests. To assess the association of neutralizing antibody titers and/or T-cell responses between levels of viremia, virus shedding and viral load in tissues, Spearman rank correlation was used. Statistical analysis and data visualization were performed using GraphPAD Prism 8.0.1(244) software (GraphPAD Software Inc., La Jolla, CA).

## Results

### Generation and *in vitro* Characterization of Recombinant SVA

**Figure 1:**
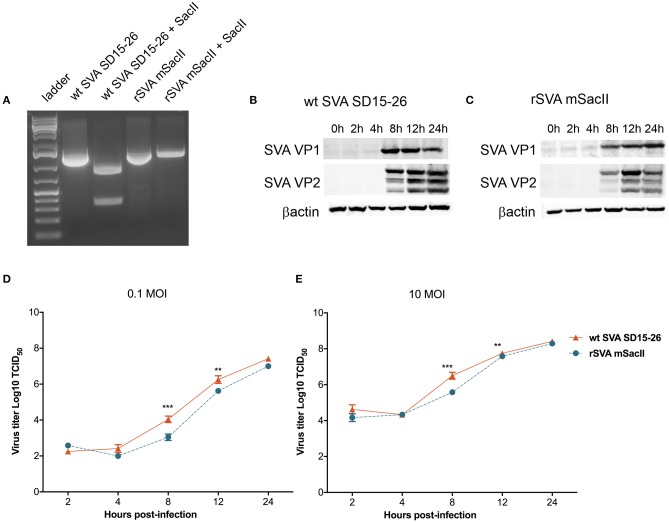
Characterization of the recombinant rSVA mSacII virus *in vitro*. **(A)** Restriction digestion with SacII enzyme of PCR amplicon of the P1 region of SVA genome. Agarose gel image shows digestion of P1-amplicon in wt SVA SD15-26 but not in rSVA mSacII. Undigested PCR products were used as controls. **(B)** Western blot to assess SVA-VP1 and VP2 protein expression in **(B)** wt SVA SD15-26 and **(C)** rSVA mSacII infected cells. H1299 cells were infected with an MOI of 10 of each virus harvested on the indicated time points and subjected to western blots using a VP1- or VP2-specific mAb. **(D)** Multi-step or **(E)** single-step growth curves. H1299 cells were infected with **(D)** 0.1 and **(E)** 10 MOI of wt SVA SD15-26 and rSVA mSacII and virus titers were determined at 2, 4, 8, 12, and 24 h post-infection; Error bars represent SEM calculated based on results of four independent experiments (*P*-values were determined by unpaired *t*-test; ^**^P < 0.01; ^***^P < 0.001).

Recently we have developed a cDNA clone for SVA strain SD15-26 (Fernandes et al., unpublished data), which was used here to develop a second recombinant SVA, originally designed to facilitate the differentiation of the rSVA virus from the parental wt SVA strain. In this clone, we introduced four additional nucleotide changes in the rSVA genome. Three changes are located in the 5′UTR (c→ t, positions 29, 31, and 32) and the fourth change consists of a silent nucleotide change (c→ a) at position 942 (VP4 coding region) of the rSVA genome (added to delete a SacII restriction endonuclease site). The identity of the rSVA mSacII was confirmed by sequencing (data not shown) and restriction digestion with SacII (Figure 1A) and its replication properties were compared to the wt SVA virus *in vitro*. Multi-step growth curves revealed a lower replication ability of the rSVA mSacII virus when compared to wt SVA, as evidenced by lower viral yields in rSVA mSacII infected cells (~1 log) between 8 and 12 h post-inoculation (*P* < 0.001) (Figures 1D,E). Additionally, expression levels of two of the main SVA capsid proteins (VP1 and VP2) were compared between wt SVA and rSVA mSacII viruses. As shown in Figures 1B,C, the levels of VP1 and VP2 protein detected in rSVA mSacII infected cells were markedly lower when compared to the levels of those proteins detected in wt SVA infected cells.

### The rSVA mSacII Virus Is Attenuated but Retains Its Immunogenicity in Pigs

**Figure 2:**
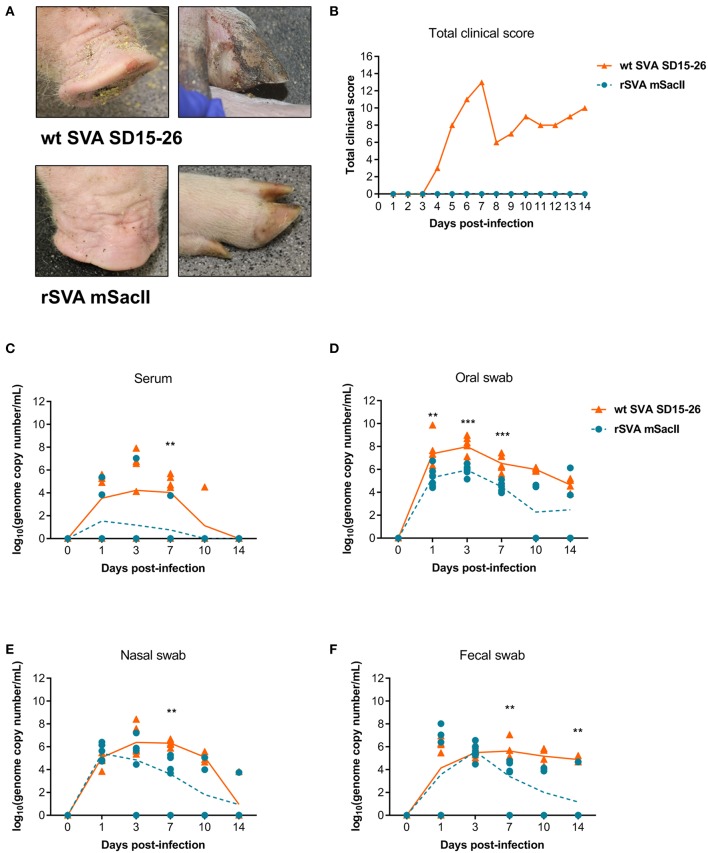
The recombinant rSVA mSacII is attenuated in swine. **(A)** Clinical outcome post-inoculation of wt SVA SD15-26 or rSVA mSacII viruses. Vesicular lesions were observed on the snout and feet of animals infected with wt SVA SD15-26 but not in animals infected with rSVA mSacII. **(B)** Total daily clinical scores post-infection in pigs. A score of 1 was attributed daily to each feet or snout presenting vesicular lesions for a total score of 5 per animal per day. **(C)** Viremia levels as determined by RT-qPCR in serum samples collected at the indicated times post-infection. Virus shedding in oral secretions **(D)**, nasal secretions **(E)** or feces **(F)** as determined by RT-qPCR on swabs collected on indicated times post-infection (*P*-values were determined by unpaired *t*-test; ^**^P < 0.01; ^***^P < 0.001).

The pathogenicity of the rSVA mSacII virus was compared to that of the wt SVA strain in pigs. Notably, while all pigs inoculated with the wt SVA strain SD15-26 presented characteristic clinical signs (lethargy, lameness) and lesions (vesicles on the snout and/or foot) of SVA infection, none of the rSVA mSacII-inoculated animals developed overt clinical disease (Figures 2A,B).

**Figure 3:**
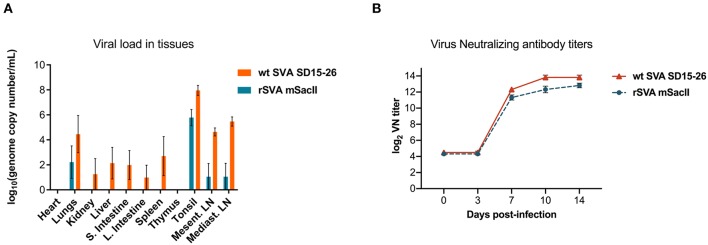
Viral load in tissues and serological responses post-infection in swine. **(A)** Viral load in tissues was determined by RT-qPCR in several tissues collected on day 14 post-infection. **(B)** Neutralizing antibody titers in both virus infected groups (Data represent group means ± SEMs).

Viremia, virus shedding (oral and nasal secretions and feces) and viral load in tissues were also evaluated. Levels of viremia and virus shedding were significantly lower in rSVA mSacII -inoculated animals when compared to wt SVA-inoculated animals (Figures 2C–F). Additionally, viral load in tissues was reduced in rSVA mSacII-inoculated animals when compared to wt SVA-inoculated animals (Figure 3A). Notably, NA responses were similar in rSVA mSacII- and wt SVA-inoculated animals (Figure 3B), indicating that the attenuated rSVA mSacII virus retained its immunogenicity in pigs.

### Clinical and Virological Findings Following Immunization With Inactivated or Live rSVA mSacII Virus

**Figure 4:**
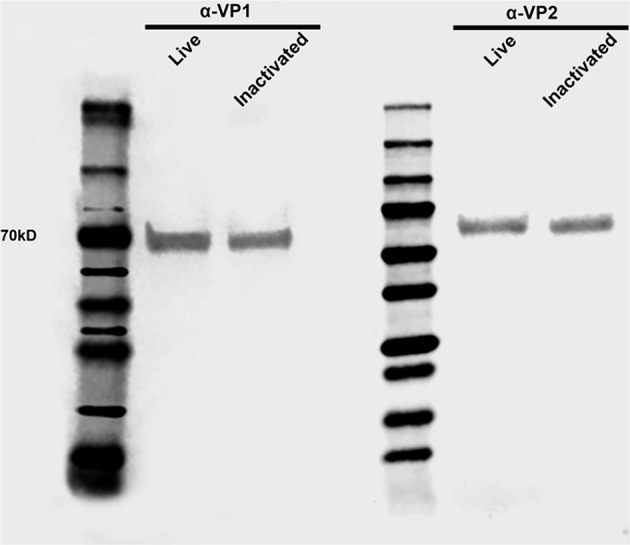
Antigen load in live and inactivated vaccine formulations. Western blot demonstrating similar levels of capsid VP1 and VP2 proteins in both live and inactivated vaccines. After dilution to a virus suspension containing 10^6^ TCID50/ml, approximately 20 μl of each vaccine preparation were subjected SDS-PAGE, transferred to a nitrocellulose membrane and probed with anti-SVA-VP1 and -VP2 specific monoclonal antibodies.

**Figure 5:**
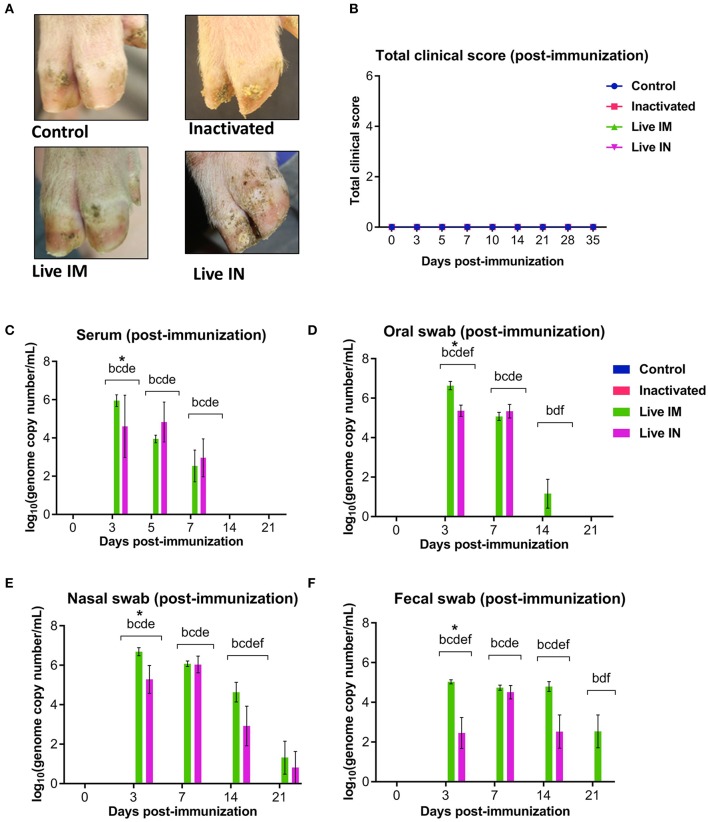
Clinical and virologic outcomes after immunization. Twenty-four 4-week old piglets were randomly allocated to four experimental groups (*n* = 6) and sham-immunized or immunized with inactivated or live (IM or IN) rSVA mSacII vaccine formulations. **(A)** Clinical outcome following immunization showing no lesions in immunized animals. **(B)** Total clinical scores following immunization. **(C)** Viremia levels determined in sera as determined by RT-qPCR at the indicated time points post-immunization. Virus shedding in oral secretions **(D)**, nasal secretions **(E)** and feces **(F)** as determined by RT-qPCR collected from immunized animals at the indicated times post-immunization. ^*^a, b, c, d, e, f indicates significant difference between groups as follows: a. Control vs. Inactivated, b. Control vs. Live IM, c. Control vs. Live IN, d. Inactivated vs. Live IM, e. Inactivated vs. Live IN and f. Live IM vs. Live IN at *P* < 0.05 (Data represent group means ± SEMs. *P*-values were determined by Tukey's multiple comparison test).

Clinical and virological parameters were evaluated following immunization of weaned piglets with inactivated or live attenuated rSVA mSacII virus. Western blot analysis of the vaccine candidate preparations demonstrated similar antigen loads for two of the major SVA capsid proteins VP1 and VP2 in both inactivated and live attenuated virus preparations (Figure 4). Following immunization, all animals were monitored daily for characteristic SVA clinical signs and vesicular lesions (22). No clinical signs nor lesions were observed in any of the immunized animals (Figure 5A). As no gross lesions were observed, clinical scores remained 0 for all groups during the post-immunization (pi) phase of the experiment (Figure 5B).

The levels of viremia were assessed in serum samples collected on days 0, 3, 5, 7, 14, and 21 pi by using a SVA real-time reverse transcriptase PCR (RT-qPCR). No viremia was detected in control (G1) and inactivated rSVA mSacII (G2)-immunized animals (Figure 5C). Whereas, SVA RNA was detected in serum from both live rSVA mSacII IM (G3) and IN (G4) immunized groups. Animals in G3 and G4 presented viremia between days 3 and 7 pi with all animals being negative from day 14 pi onwards (Figure 5C).

Virus shedding was assessed in oral and nasal secretions and feces. Oral, nasal and rectal swabs collected on days 0, 3, 5, 7, 14, and 21 pi were tested by RT-qPCR. Virus shedding was detected up to day 14 pi on oral secretions or up to day 14–21 pi on nasal secretions and feces of animals in the live IM (G3) and live IN (G4)-vaccine groups, respectively (Figures 5D–F). No virus excretion was detected on control (G1) and inactivated (G2) groups (Figures 5D–F).

### Immunogenicity of Inactivated or Live rSVA mSacII Virus in Pigs

**Figure 6:**
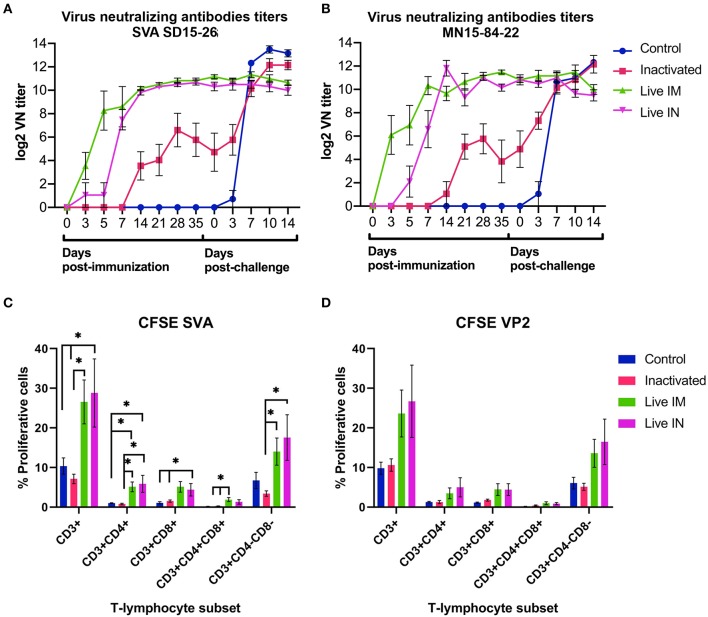
Immune responses elicited by immunization and/or challenge infection. **(A,B)** Virus neutralizing antibody responses against SVA strain SD15-26 **(A)** or MN15-84-22 **(B)** as determined by VN assays performed in serum samples collected at the indicated times post-immunization and post-challenge. **(C)** Carboxyfluorescein succinimidyl ester (*CFSE*) proliferation assay performed in PBMCs obtained on day 42 pi (day of challenge). Cells were stimulated with UV-inactivated SVA (MOI = 1) for 5 days and proliferative responses of major swine T cell subsets were determined by flow cytometry. **(D)** CFSE proliferation assay performed in PBMCs obtained on day 42 pi (day of challenge). Cells stimulated with recombinant SVA VP2 protein (1 μg/mL) for 5 days and proliferative responses of major swine T cell subsets were determined by flow cytometry. Proliferative T cells were expressed as percent of total CD3^+^ T cells on each sample (Data represent group means ± SEMs. *P*-values were determined by Tukey's multiple comparison; ^*^P < 0.05).

The immunogenicity of inactivated or live rSVA mSacII virus was evaluated. Humoral immune responses were assessed by virus neutralization assays (9, 19) in serum samples collected on days 0, 3, 5, 7, 14, 21, 28, and 35 pi. Immunization with live rSVA mSacII virus via the IM (G2) and IN (G3) routes, elicited robust NA responses with high antibody titers being detected as early as day 3 pi in animals in the live IM group (G3) (Figures 6A,B). Interestingly, early NA titers elicited by live IM immunization were significantly higher than antibody titers elicited by IN immunization (day 5–7 pi; *P* < 0.01), whereas no differences in NA titers in the live IM and IN immunized groups were observed after day 14 pi (Figures 6A,B). Notably, a single dose of the live rSVA mSacII administered either by the IM or IN routes resulted in significantly higher NA responses when compared to immunization with the inactivated virus formulation, even after the booster immunization on day 21 pi (Figures 6A,B, days 3–35 pi; *P* < 0.01). No NA antibodies were detected in control sham-immunized animals (G1) prior to challenge infection (days 0–35 pi) (Figures 6A,B).

T cell responses elicited by immunization with rSVA mSacII virus were evaluated by lymphocyte proliferation assays. Peripheral blood mononuclear cells (PBMCs) collected on day 42 pi (day of challenge) were subjected to *in vitro* recall stimulation with SVA or with recombinant SVA-VP2 protein as previously described (19). Interestingly, significant recall T cell (CD3^+^) proliferation was detected in animals immunized with live rSVA mSacII via the IM (G3) and IN (G4) routes (*P* < 0.05) (Figure 6C). Additionally, proliferative recall responses of individual T cell subsets, including CD4+, CD8^+^, double positive CD4^+^/CD8^+^ and γδ T cells (double negative CD4^−^/CD8^−^ cells) were also significantly higher in animals in the live IM (G3) and live IN (G4) groups (*P* < 0.05) (Figure 6C). A similar trend in recall T cell proliferation was observed in animals immunized with live virus and re-stimulated with recombinant VP2 protein (Figure 6D).

### Immunization With rSVA mSacII Protects Against Heterologous SVA Challenge

**Figure 7:**
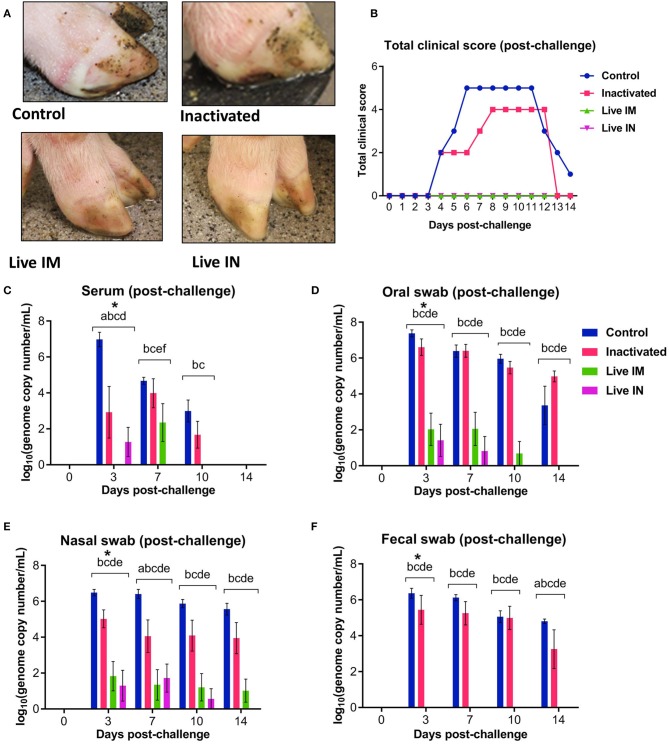
Clinical and virologic outcomes after heterologous SVA challenge. Animals in all groups were challenged oronasally with SVA MN15-84-22 (10^8.5^ TCID_50_). **(A)** Clinical outcome following immunization showing no lesions in immunized animals. **(B)** Total clinical scores following immunization. **(C)** Viremia levels determined in sera by RT-qPCR at the indicated time points post-immunization. Virus shedding in oral secretions **(D)**, nasal secretions **(E)** and feces **(F)** as determined by RT-qPCR at the indicated times post-challenge. ^*^a, b, c, d, e, f indicates significant differences between groups as follows: a. Control vs. Inactivated, b. Control vs. Live IM, c. Control vs. Live IN, d. Inactivated vs. Live IM, e. Inactivated vs. Live IN and f. Live IM vs. Live IN at *P* < 0.05 (Data represent group means ± SEMs. *P*-values were determined by Tukey's multiple comparison).

The protective efficacy of inactivated or live rSVA mSacII virus were evaluated following challenge infection with a heterologous contemporary SVA strain. All immunized animals were challenged oronasally with a virulent SVA strain SVA MN15-84-22 (9) on day 42 post-immunization (Table 1). All animals in control sham-immunized G1 presented clinical signs and/or lesions of SVA starting on day 4 post-challenge (pc). Animals presented lethargy and lameness and four of six animals (4/6; 66.6%) displayed characteristic vesicular lesions (Figure 7A). Additionally, 3/6 (50%) animals in the inactivated G2 developed lameness and characteristic VD (Figure 7A). Similar to control animals, G2 animals developed lesions on or after day 4 pc (Figures 7A,B). Notably, a single dose of the live rSVA mSacII via the IM (G3) or the IN (G4) routes resulted in protection from clinical SVA, as no clinical signs nor lesions were observed in immunized animals (Figures 7A,B). Peak clinical scores were observed on day 6 pc in control animals or on day 8 pc in the inactivated vaccine group (Figure 7B). As no gross lesions were observed in both live vaccine groups (G3 and G4) (Figure 7A), clinical scores for these groups remained 0 throughout the challenge phase of the experiment (Figure 7B).

The levels of viremia were also assessed post-challenge infection. Serum samples collected on days 0, 3, 7, 10, and 14 pc were tested for SVA RNA by RT-qPCR. SVA viremia was detected in control sham-immunized (G1) and in the inactivated group (G2) animals between days 3 and 10 pc (Figure 7C). The levels of viremia in animals immunized with live rSVA mSacII IM and IN were significantly lower than in control- and inactivated rSVA mSacII-immunized animals (Figure 7C, *P* < 0.01). In fact, only 2/6 animals immunized with live rSVA mSacII IM and 3/6 immunized IN presented viremia on day 3 or 7 pc, respectively (data not shown).

Virus shedding was assessed in oral and nasal secretions and feces in swabs collected on days 0, 3, 7, 10, and 14 pc by RT-qPCR. High levels of virus excretion were detected between days 3 and 14 pc in animals from the control (G1) and inactivated-vaccine groups (G2) (Figures 7D–F). Virus shedding was significantly lower in animals from live IM (G3)- and live IN (G4) groups (*P* < 0.01) when compared to both control (G1) and inactivated (G2) groups in oral and nasal secretions (Figures 7D,E). Interestingly, no virus shedding was detected in feces in the live IM (G3)- and live IN (G4) group animals (Figure 7F).

**Figure 8:**
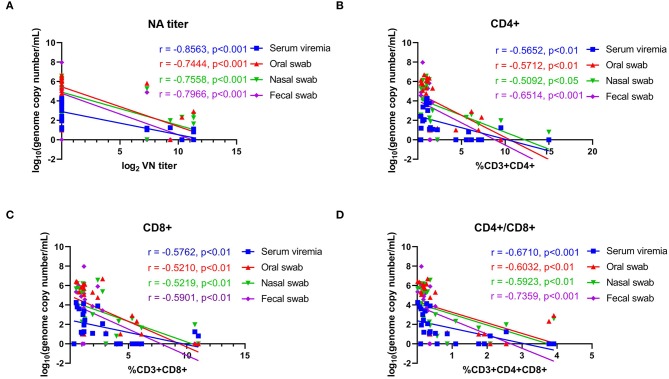
Correlations between neutralizing antibody titers and proliferative T-cell responses with viremia and virus shedding. **(A)** Correlation between the levels of neutralizing antibody (NA) titers at day 42 pi (day of challenge) with levels of viremia, virus shedding in oral secretions, nasal secretions and feces with post challenge infection (days 43–63 pi). **(B)** Correlation of CD4+ T-cell proliferation at day 42 pi (day of challenge) with levels of viremia, virus shedding in oral secretions, nasal secretions and feces post challenge infection (days 43–63 pi). **(C)** Correlation of CD8^+^ T-cell proliferation at day 42 pi (day of challenge) with the levels of viremia, virus shedding in oral secretion, nasal secretion and fecal swab post challenge infection (days 43–63 pi). **(D)** Correlation of CD4^+^CD8^+^ T-cell proliferation at day 42 pi (day of challenge) with the levels of viremia, virus shedding in oral secretions, nasal secretions and feces post challenge infection (days 43–63 pi). Groups included in the analysis are Control, Live IN, Live IN, and Inactivated. Correlation coefficient (R) values and statistics for each correlation are shown in the graphs (95% CI).

The association of NA responses (day 42 pi/0 pc) and levels of viremia/virus shedding were evaluated in samples collected from control and immunized animals. Data points used in the correlation analysis included samples from day 1 through 14 pc. A high negative correlation between NA levels and levels of viremia (*r* = −0.856, 95% CI = −0.938 to −0.685, *P* < *0.001*), virus shedding in oral (*r* = −0.744, 95% CI = −0.885 to −0.478, *P* < *0.001*), nasal secretions (*r* = −0.756, 95% CI = −0.891 to −0.497, *P* < *0.001*), and feces (*r* = −0.797, 95% CI = −0.910 to −0.571, *P* < *0.001*) was observed (Figure 8A). We also assessed the correlations between T-cell responses and levels viremia/virus shedding. Low to moderate negative correlation between proliferative CD4^+^ and CD8^+^ T cell responses and levels of viremia/virus shedding (oral, nasal and fecal) was observed (Figure 8B). Additionally, moderate to high negative correlation between proliferative double positive CD4^+^CD8^+^ T cell responses and viremia/virus shedding was detected (Figures 8B–D).

### Neutralizing Antibody Responses Post-challenge Infection

The serological responses post-challenge infection was evaluated by VN assays. Serum samples collected on days 0, 3, 7, and 14 pc were tested by VN assays. As shown in Figures 6A,B, all animals in control (G1) and inactivated (G2) groups seroconverted post-SVA challenge infection, presenting an anamnestic increase in NA antibody titers. Levels of NA detected in control animals on days 7, 10, and 14 pc were significantly higher than in inactivated (G2), live IM (G3), and live IN (G4) groups (Figures 6A,B). Notably, no increase in NA titers were detected in animals from the live IM (G3) and live IN (G4) groups after challenge infection (Figures 6A,B).

### Protective Responses Elicited by Immunization With rSVA mSacII Lead to Decreased Viral Load in Tissues

**Figure 9:**
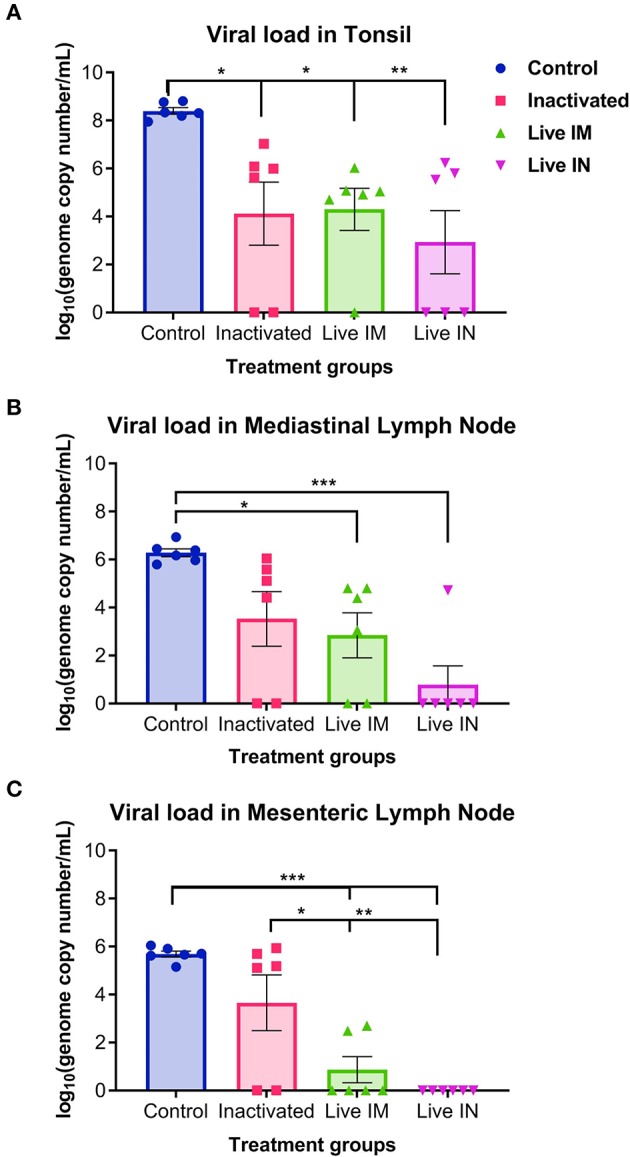
Viral load in tissues. Virus load in the **(A)** tonsil, **(B)** mediastinal lymph node, and **(C)** mesenteric lymph node as determined by RT-qPCR on tissues collected at necropsy on day 14 post-challenge (Data represent group means ± SEMs. *P-*values were determined by Tukey's multiple comparison; ^*^P < 0.05; ^**^P < 0.01; ^***^P < 0.001).

Viral load was assessed in lymphoid tissues (tonsil, mediastinal and mesenteric lymph nodes) following challenge infection (day 14 pc) using RT-qPCR. Immunization with inactivated or live rSVA mSacII virus led to a marked decrease in viral load in all tissues tested (Figure 9). Significantly lower SVA genome copy numbers were detected in the tonsil of animals immunized with inactivated (G2, *P* < 0.05)-, live IM (G3, *P* < 0.05), or live IN (G4, *P* < 0.05) rSVA mSacII virus, when compared to control animals (G1) (Figure 9A). No significant differences in SVA RNA copy number was observed between inactivated (G2)-, live IM (G3)-, live IN (G4)- rSVA mSacII immunized animals (Figure 9A). Viral load in mediastinal or mesenteric lymph nodes were significantly lower in live IM (G3; *P* < 0.05)-, live IN (G4, *P* < 0.001)- rSVA mSacII immunized animals when compared to control animals (G1) (Figures 9B,C). The levels of SVA load in tissues of animals in the live IN (G4) group were lower with fewer animals being positive in all three tissues when compared to animals in G2 and G3 (Figure 9).

**Figure 10:**
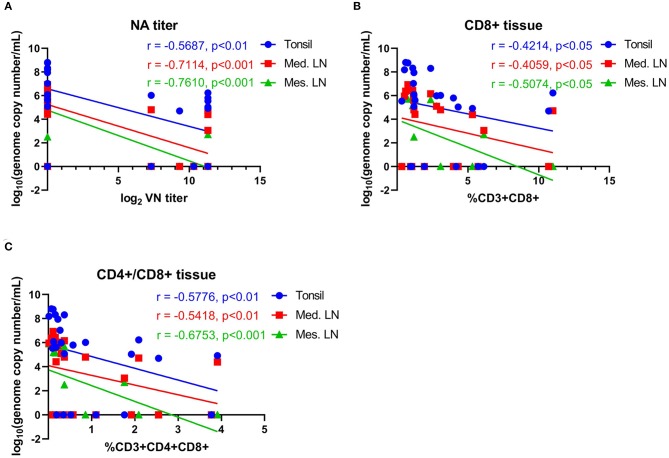
Correlations between neutralizing antibody titers and T-cell responses with viral load in tissues. **(A)** Correlation between NA antibody titers at day 42 pi (day of challenge) with viral load in tonsil, mediastinal lymph node and mesenteric lymph node measured on day 63 pi (day of necropsy). **(B)** Correlation of CD8^+^ T-cell proliferation at day 42 pi (day of challenge) with viral load in tonsil, mediastinal lymph node and mesenteric lymph node on day 63 pi (day of necropsy). **(C)** Correlation of CD4^+^CD8^+^ T-cell proliferation at day 42 pi (day of challenge) with viral load in tonsil, mediastinal lymph node, and mesenteric lymph node on day 63 pi (day of necropsy). Groups included in the analysis are Control, Live IN, Live IN, and Inactivated. Correlation coefficient (R) value and statistics for each correlation are shown in the graphs (95% CI).

The association between NA- and T cell responses with viral load in tissues (day 14 pc) was evaluated. Moderate-to-high negative correlations between NA levels and vial load in the tonsil (*r* = −0.569, 95% CI = −0.795 to −0.202, *P* < *0.01*), mediastinal LN (*r* = −0.711, 95% CI = −0.869 to −0.422, *P* < *0.001*), and mesenteric LN (*r* = −0.761, 95% CI = −0.894 to −0.507, *P* < *0.001*) were observed (Figure 10A). We also observed an association between T cell responses and tissue viral load. Moderate negative correlation between CD8+ and CD4+CD8+ double positive T cells were observed (Figures 10B,C).

## Discussion

Here we generated an attenuated rSVA strain and assessed its safety and efficacy when administered as an inactivated/adjuvanted or live attenuated vaccine against heterologous SVA challenge in pigs. The rSVA mSacII was generated using reverse genetics and engineered to contain three nucleotide changes in the 5′UTR region (C→ T) of the genome and one silent nt change (C→ A) in the P1/VP4 coding region. The three nucleotides substitutions in the 5′UTR region (C→ T) were derived from the low virulence SVA strain SVV001, while the change in the P1/VP4 region (position 942) was inserted to delete a SacII restriction site from the virus genome.

Although originally designed with the intent of rescuing a virulent rSVA strain, *in vitro* characterization of the rSVA mSacII virus demonstrated lower viral yields and protein expression in infected cells when compared to the wt SVA SD15-26 virus. Most importantly, inoculation of finishing pigs with the rSVA mSacII virus did not result in overt VD, and inoculated animals presented lower levels of viremia and virus shedding in oral, nasal secretions and feces when compared to animals inoculated the wt SVA SD15-26 virus. These findings indicated that the rSVA mSacII virus was attenuated in pigs. The mechanism(s) of attenuation of rSVA mSacII was/were not investigated in our study, however, it is possible that the nucleotide changes introduced in the 5′UTR and/or in the P1 coding region may have affected the conformation of RNA secondary structures present in these regions of the virus genome [e.g., internal ribosomal entry site [IRES] or cis-active RNA elements [CRE], respectively] (26–28). Changes affecting the conformation of these RNA structures have been linked to impaired protein expression and/or picornavirus replication, thus resulting in decreased virus virulence and attenuated disease phenotype (29–31). Results here showing lower viral yields and reduced protein expression levels in rSVA mSacII infected cells support this hypothesis. However, additional studies are needed to dissect the precise molecular determinants that led to attenuation of the rSVA mSacII virus in pigs. Notably, despite its attenuated phenotype no significant differences in the levels of NA were observed between animals inoculated with rSVA mSacII or with the parental wt SVA SD15-26, demonstrating that rSVA mSacII virus retained its immunogenicity.

Given the attenuated phenotype and the immunogenicity of the rSVA mSacII in pigs, the next step of our study was to assess the potential of this viral strain as a vaccine candidate for SVA. Previously we have shown that both antibody- and T cell responses are correlated with the control of SVA infection and peak antibody and T cell responses parallel with disease resolution (19). Thus, here we assessed the efficacy of inactivated- or live rSVA mSacII vaccine formulations against a heterologous SVA challenge. Following immunization none of the animals presented clinical signs nor lesions of SVA, confirming efficient inactivation- (G2) and, most importantly, attenuation of the rSVA mSacII virus (G3 and G4). As expected, animals immunized with the live rSVA mSacII via the IM or IN routes excreted virus in nasal and oral secretions and in feces for 7–21 days post-immunization. Restriction enzyme (Sac II) analysis of the P1 region from PCR amplicons obtained directly from serum and/or nasal secretions from immunized animals on day 3 post-immunization confirmed the identity of the rSVA mSacII sequences during replication of the vaccine virus in pigs (data not shown). These results indicate that rSVA mSacII is a good vaccine candidate to prevent SVA in swine. Additional studies are required, however, to evaluate the genetic stability of this virus after passage/transmission among a cohort of immunized/commingled animals.

The immune responses elicited by immunization with inactivated or live rSVA mSacII vaccine formulations were also evaluated. Notably, while robust neutralizing antibody responses were detected in all animals immunized with a single dose of the live rSVA mSAcII via the IM and the IN routes, animals immunized with the inactivated vaccine formulation presented a delayed NA response with several animals only seroconverting after the booster immunization on day 21 pi. Importantly, one dose of the live rSVA mSacII virus elicited significantly higher NA responses against SVA, than two doses of the inactivated rSVA mSacII vaccine formulation. Although a secondary humoral immune response was observed after the booster immunization with the inactivated rSVA mSacII vaccine, NA titers never reached the levels elicited by immunization with the live virus. This is likely a result of antigen amplification during replication of the live attenuated virus in immunized animals leading to a broader and more efficient stimulation of the immune system. This was also evident at the T cell level, as recall stimulation of PBMCs from animals immunized with the live rSVA mSacII led to robust proliferative responses of CD4^+^, CD8^+^, CD4^+^CD8^+^, and γδ- T cells (CD4^−^CD8^−^).

The association between the capacity of picornavirus vaccines to elicit virus-specific immune responses and protection against challenge infection has been reported for several picornaviruses (23, 24, 32, 33). Neutralizing antibodies seem to be correlated with protection against most picornaviruses (23, 33–35), while the role of T cell responses is debatable with some studies showing the contribution of T cells to protection (36), while others demonstrate only partial protection (37). The protective efficacy of the rSVA mSacII vaccine formulations and the immune responses elicited by immunization with these vaccine candidates was investigated here following challenge of immunized animals with a virulent heterologous SVA strain (MN15-84-22 at 10^8.5^ TCID_50_; day 42 post-immunization) (Table 1). While 4 out of 6 (4/6) animals in the control group and 3/6 animals in the inactivated vaccine group presented characteristic SVA lesions, none of the animals in the live IM and IN groups presented clinical signs or lesions compatible with SVA infection. Consistent with the clinical outcome post-challenge, the magnitude and extent of viremia and virus shedding in nasal and oral secretions and feces was significantly lower in animals immunized with the live rSVA mSacII virus via the IM or IN routes. Notably, no fecal excretion was detected in the live IM and IN groups following challenge infection. Additionally, no anamnestic serological responses were detected in animals in the IM and IN after the challenge infection, whereas control animals and animals in the inactivated vaccine group seroconverted to the challenge virus. Together, these findings demonstrate solid protection of animals immunized with the live attenuated rSVA mSacII virus against heterologous virus challenge. Immunization with the inactivated rSVA mSacII formulation, on the other hand, only elicited partial protection. These contrasting protective efficacies may potentially reflect significant differences in the levels of neutralizing antibodies and/or T cell responses elicited by live or inactivated virus immunization (Figure 6).

Recently the immunogenicity and protective efficacy of a cell culture derived inactivated SVA vaccine candidate was evaluated in pigs (23). This study demonstrated that animals immunized with a 2 μg-dose of the vaccine formulation developed higher titers of NA antibodies and were protected against homologous SVA challenge (23). Animals that received 1/3 or 1/9 of the vaccine dose, however, developed lower levels of NA antibodies and were only partially protected against challenge infection (23). Although a parallel between the levels/titers of neutralizing antibodies elicited by immunization and protection was observed in the present study and in the study by Yang and collaborators (23), studies with other picornaviruses, including FMDV, have shown that this correlation is not precise (23, 34, 37). While some animals presenting low NA titers can resist challenge infection, others will succumb and develop overt clinical disease (23, 34). Thus, defining protective antibody levels for picornaviruses is complex and it is complicated by the role of antibodies in opsonization and phagocytosis and by the potential involvement of T cells in protection against virus infection (23, 38).

Viral load in tissues was also evaluated in our study as a measure of vaccine elicited protection. For this, SVA RNA present in lymphoid tissues, including tonsil and mediastinal- and mesenteric lymph nodes was quantified by RT-qPCR. These tissues have been shown to harbor SVA with the tonsil potentially serving as one of the primary sites for virus replication (18, 22). Results here show low amounts of SVA RNA in tonsil and mediastinal and mesenteric lymph nodes in animals from the live vaccine groups (Figure 9). Some animals did not present detectable levels of SVA RNA in the tonsil and many animals did not present detectable viral RNA in the lymph nodes. A lower frequency of positive animals was detected in the group immunized with the live rSVA mSacII via the IN route, suggesting a more effective role of local mucosal immunity in protection against SVA challenge when compared to systemic immunity elicited by parenteral administration of the inactivated or live vaccine formulations. The lower amounts of virus detected in tissues of animals in the live IM or live IN vaccine groups when compared to the viral load detected in inactivated group, may also be a result of increased T cell activity and virus clearance elicited by the live virus vaccine. Additional studies are needed, however, to characterize local mucosal immunity following IN immunization with SVA and to dissect the function of T cells in protection against SVA.

This study describes the development of an effective live attenuated rSVA vaccine candidate capable of providing solid protection against heterologous SVA challenge in pigs. A single dose of the live attenuated vaccine candidate administered via the IM or IN routes elicited protection to challenge with a virulent SVA strain, as evidenced by lack of clinical signs and lower levels of viremia, virus shedding and viral load in tissues. Given that currently there is only one known SVA serotype circulating in the swine population worldwide (22), the live attenuated vaccine candidate developed here may represent a valuable tool to prevent and control SVA outbreaks.

## Data Availability Statement

All datasets generated for this study are included in the manuscript.

## Ethics Statement

All animal studies were carried out in accordance with the principles of the Animal Welfare Act, and the recommendations of the Guide for the Care and Use of Agricultural Animals in Research and Teaching. The protocols were approved by the SDSU IACUC (16-002A and 18-032A).

## Author Contributions

BS, MF, and DD contributed conception and design of the study, and data analysis. ML contributed in the design of the vaccine candidate. BS and LJ performed critical experiments and statistical analysis. SL contributed with animal experiments. BS and DD wrote the manuscript. All authors contributed to manuscript revision, read, and approved the submitted version.

### Conflict of Interest

A provisional patent application has been submitted to the United States Patent and Trademark Office for this the vaccine candidate (US serial no. 62/874,094). The authors declare that the research was conducted in the absence of any commercial or financial relationships that could be construed as a potential conflict of interest.
